# 2170. Association Between End-of-Treatment Procalcitonin Levels with Mortality & Recurrent Ventilator-Associated Pneumonia

**DOI:** 10.1093/ofid/ofac492.1790

**Published:** 2022-12-15

**Authors:** Sam Schuiteman, Owen Albin

**Affiliations:** University of Michigan, Ann Arbor, Michigan; University of Michigan Medical School, Ann Arbor, Michigan

## Abstract

**Background:**

In the only randomized controlled trial utilizing procalcitonin in ventilator-associated pneumonia (VAP), 30% of patients failed to normalize procalcitonin levels by the end of treatment, a finding of uncertain clinical importance. The prognostic value of procalcitonin for VAP mortality has been examined only in studies with limited sample sizes and the relationship between end-of-treatment procalcitonin (EOT-P) and VAP recurrence—which affects up to 40% of patients with VAP—has never been examined. We aimed to determine the relationship between VAP EOT-P and recurrent pneumonia or mortality.

**Methods:**

Retrospective single-center cohort study of hospitalized adult patients between 2013 – 2022 with VAP (defined as use of invasive mechanical ventilation for ≥ 2 days, positive respiratory culture and treatment for ≥ 5 days with pneumonia-specific antibiotics) who had serum procalcitonin levels obtained within 48 of antibiotic completion (AC). Exclusion criteria included death or discharge to hospice within 48 hours of AC. Patients with EOT-P < 0.5 were compared with those with EOT-P ≥ 0.5. The primary outcome was a composite endpoint of recurrent pneumonia (defined as clinical suspicion sufficient to warrant respiratory culture collection) and/or death within 30 days of AC.

**Results:**

Of 140 included patients, 79 (56.4%) had EOT-P levels < 0.5, and 61 (43.6%) had levels ≥ 0.5. Patients with elevated EOT-P were more likely to have renal disease, longer duration of antibiotic treatment and longer antecedent hospital length of stay than those with non-elevated EOT-P. Demographic characteristics and comorbidities were otherwise similar between groups (Table 1). After multivariable adjustment, patients with elevated EOT-P were significantly more likely to have recurrent pneumonia or death within 30 days (Table 2 and Figure 1, OR 2.39 (95% 1.19-4.80 CI), adjusted OR 2.37 (95% CI 1.09-5.17)).

**Figure:**
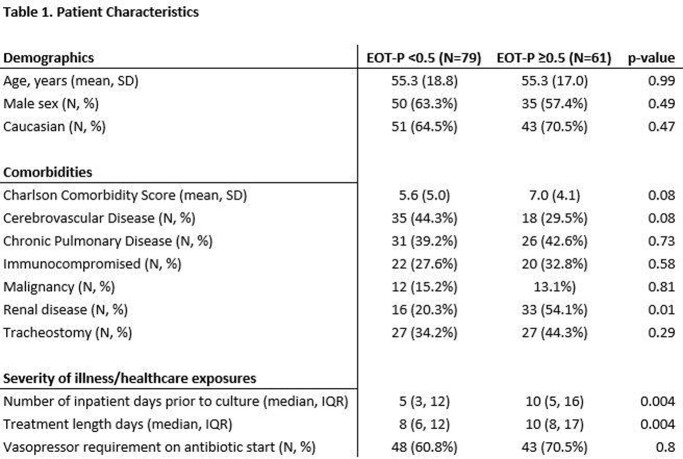

Patient demographics of the study group.

"EOT-P" = serum procalcitonin level drawn following completion of antibiotics. Patients with EOT-P <0.5 were compared with those with EOT-P ≥ 0.5.

"Immunocompromised" = combination of HIV/AIDS positivity, actively taking immunosuppressive medications, metastatic or hematologic malignancy, history of stem cell transplant, or organ transplantation.

**Figure:**
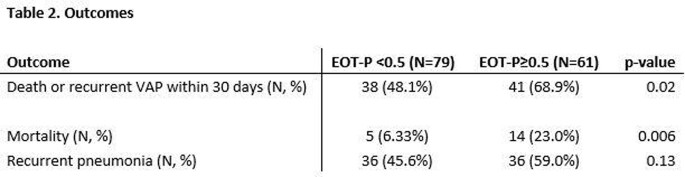

Patient outcomes, followed for 30 days following antibiotic completion. The primary endpoint was a composite of death or recurrent pneumonia. The secondary endpoints are death or recurrent pneumonia examined separately.

"EOT-P" = serum procalcitonin level drawn following completion of antibiotics. Patients with EOT-P <0.5 were compared with those with EOT-P ≥ 0.5.

"VAP" = ventillator-assisted pneumonia.

**Figure:**
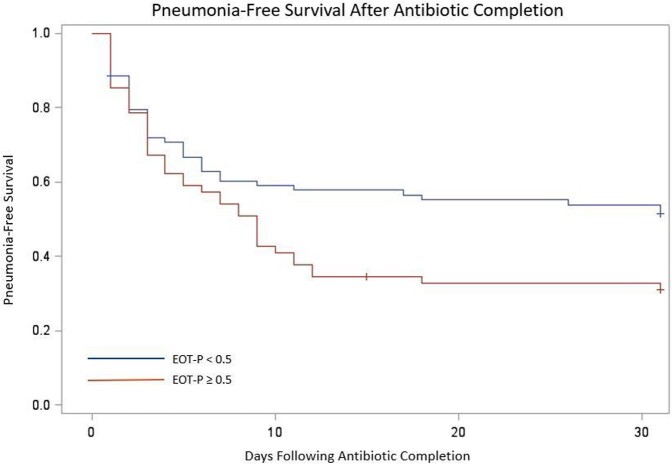

Percentage of patients who survived without recurrence of pneumonia, followed for 30 days following antibiotic completion.

"EOT-P" = serum procalcitonin level drawn following completion of antibiotics. Patients with EOT-P <0.5 were compared with those with EOT-P ≥ 0.5.

**Conclusion:**

Elevated EOT-P in VAP was independently associated with increased VAP recurrence or mortality within 30 days. To our knowledge, this is the largest study to date of the prognostic value of procalcitonin in a ventilator-associated pneumonia cohort. Use of end-of-treatment procalcitonin in VAP may serve as a candidate biomarker to predict VAP recurrence and death.

**Disclosures:**

**Owen Albin, MD**, Charles River Laboratory: Advisor/Consultant|Cipla Pharmaceuticals: Advisor/Consultant|Shionogi Inc: Advisor/Consultant.

